# IL-1*β*-Induced Matrix Metalloprotease-1 Promotes Mesenchymal Stem Cell Migration via PAR1 and G-Protein-Coupled Signaling Pathway

**DOI:** 10.1155/2018/3524759

**Published:** 2018-04-05

**Authors:** Ming-Siou Chen, Cheng-Yu Lin, Yun-Hsuan Chiu, Chie-Pein Chen, Pei-Jiun Tsai, Hwai-Shi Wang

**Affiliations:** ^1^Institute of Anatomy and Cell Biology, School of Medicine, National Yang-Ming University, Taipei, Taiwan; ^2^Division of High Risk Pregnancy, Mackay Memorial Hospital, Taipei, Taiwan; ^3^Department of Critical Care Medicine, Taipei Veterans General Hospital, Taipei, Taiwan

## Abstract

Mesenchymal stem cells (MSCs) are known for homing to sites of injury in response to signals of cellular damage. However, the mechanisms of how cytokines recruit stem cells to target tissue are still unclear. In this study, we found that the proinflammation cytokine interleukin-1*β* (IL-1*β*) promotes mesenchymal stem cell migration. The cDNA microarray data show that IL-1*β* induces matrix metalloproteinase-1 (MMP-1) expression. We then used quantitative real-time PCR and MMP-1 ELISA to verify the results. MMP-1 siRNA transfected MSCs, and MSC pretreatment with IL-1*β* inhibitor interleukin-1 receptor antagonist (IL-1RA), MMP tissue inhibitor of metalloproteinase 1 (TIMP1), tissue inhibitor of metalloproteinase 2 (TIMP2), MMP-1 inhibitor GM6001, and protease-activated receptor 1 (PAR1) inhibitor SCH79797 confirms that PAR1 protein signaling pathway leads to IL-1*β*-induced cell migration. In conclusion, IL-1*β* promotes the secretion of MMP-1, which then activates the PAR1 and G-protein-coupled signal pathways to promote mesenchymal stem cell migration.

## 1. Introduction

Umbilical cord mesenchymal stem cells possess several properties that make them of interest as a source of cells for therapeutic use [1]. Stem cells migrating toward damaged tissues play critical roles in wound healing and tissue regeneration [2]. It was assumed that tissue damage or apoptosis releases factors that recruit stem cells to the damaged site, where the mobilized stem cells then proliferate and differentiate to replace damaged tissues [3, 4]. It has been found that systematically infused mesenchymal stem cells possess the ability to migrate to sites of injured or inflamed tissues and exert therapeutic effects [5]. However, the mechanisms involved in the homing functions of stem cells are still not fully understood. Recent research has shown that inflamed and ischemic tissue may release cytokines or growth factors such as stromal cell-derived factor- (SDF-) 1*α*, transforming growth factor- (TGF-) *β*1, monocyte chemotactic protein- (MCP-) 1, tumor necrosis factor- (TNF-) *α*, and interleukins (IL) to promote mesenchymal stem cells homing to the injured region [2, 6]. Several cytokines and growth factor receptors are found in mesenchymal stem cells, including interleukin-1 receptor (IL-1R) [7].

Interleukin-1*β* plays an important role in inflammation and tissue damage in many organs. IL-1*β* is involved in a range of cellular functions, including cell proliferation, differentiation, and apoptosis. IL-1*β* also induces cell migration and homing by activating downstream protein kinase cascades, which leads to the expression of inflammatory proteins [8]. Furthermore, it has been observed that IL-1*β* enhances lymphocyte and eosinophil cell adhesion and transendothelial migration [9, 10]. Some studies have reported that IL-1*β* is capable of inducing different types of matrix metalloproteinase (MMP) expressions, which can degrade extracellular matrix and promote cell migration [8, 11–14]. It has been reported that IL-1*β*-induced MMP-1 expression in chondrocytes involved ERK activation [15].

Cellular migration is a complex process encompassing the disintegration of extracellular matrix, detachment of cells from the basal membrane, migration of cells from the original location, intravasation into the target tissue, and interaction with the target microenvironments [16]. The breakdown of the extracellular matrix requires the action of proteolytic enzymes such as MMPs, which are zinc-dependent endopeptidases [17]. MMPs are secreted as inactive proenzymes or zymogens, which are activated by the cleavage of the prodomain [18]. Depending on the substrate specificity and structure, MMPs are divided into several subgroups: collagenases (e.g., MMP-1), gelatinases (e.g., MMP-2 and MMP-9), stromelysins (e.g., MMP-3 and MMP-10), matrilysins (e.g., MMP-7 and MMP-26), and membrane-type matrix metalloproteinase-1 (MT1-MMP). In particular, interstitial collagenase (MMP-1) has been found to be involved in the invasion of breast carcinoma [19]. Stromal-derived MMP-1 have been shown to cleave and activate G-protein-coupled receptor PAR1, leading to the activation of intracellular signals regulating the invasion process in breast cancer cells [20]. Recently, the functional role of MMP-1 in the migratory activities of stem cells was studied. Ho et al. [21] found that targeted knockdown of endogenous MMP-1 inhibits the migration ability of mesenchymal stem cells in vitro.

Previous studies demonstrated that inflammatory cytokines such as transforming growth factor- (TGF-) *β*1, tumor necrosis factor- (TNF-) *α*, and IL-1*β* increase the production of MMPs in stem cells, resulting in a strong stimulation of chemotactic migration through the extracellular matrix [2, 22]. These findings indicate that enhancement of the homing capacity of stem cells can be achieved through the modulation of mesenchymal stem cell responses to a variety of growth factors and cytokines.

Protease-activated receptor (PAR) 1 is a G-protein-coupled receptor identified with the discovery of the first thrombin receptor [23, 24]. PAR1 activation by thrombin and other trypsin-like serine-like proteases is based on protease cleaving of the N-terminal domain of the receptor and the release of a tethered ligand binding to an extracellular loop of the receptor, subsequently activating the G-protein-coupled signal transduction [25]. PAR1 plays a central role in tissue repair, fibrosis, inflammation, neurodegeneration, atherosclerosis, and restenosis [26–28]. It has been reported that MMP-1 performs an important role in tumor progression by activating PAR1 [20]. Additionally, PAR1 has been found to be involved in the invasive and metastatic processes of cancers of the breast, colon, lung, pancreas, prostate, and melanoma [20, 29–32]. Furthermore, Ho et al. [21] reported that the interference of interaction between MMP-1 and PAR1 seriously reduced the migration capability of stem cells, indicating the importance of the MMP-1-PAR1 signaling axis in regulating the migration ability of mesenchymal stem cells.

In this study, we demonstrated that proinflammation cytokine IL-1*β* promotes mesenchymal stem cell migration, which can be inhibited by IL-1RA. Furthermore, we found that IL-1*β* can increase MMP-1 secretion [33]. As a result of the inhibition of MMP-1 secretion by TIMP1, TIMP2, and MMP-1 inhibitor GM6001 and MMP-1 siRNA transfection, PAR1 activation and stem cell migration were inhibited. By using IL-1RA (IL-1*β* inhibitor) and SCH79797 (PAR1 inhibitor), the migration ability of stem cells was also decreased. Taken together, we are of the opinion that IL-1*β*-mediated stem cell migration involves MMP-1 expression, which then activates PAR1 and finally influences mesenchymal stem cell migration via its signaling transduction pathway.

## 2. Materials and Methods

### 2.1. Cell Culture

Human umbilical cord mesenchymal stem cells were maintained in low-serum defined medium: 56% low-glucose Dulbecco's modified Eagle medium (Invitrogen, CA, USA), 37% MCDB 201 (Sigma, MO, USA), 2% fetal bovine serum (Thermo, Logan, UT), 0.5 mg/ml albumin (Sigma, MO, USA), 1x insulin-transferrin-selenium-A (Invitrogen, CA, USA), 1x antibiotic antimycotic solution (Thermo, Logan, UT), 10 nM dexamethasone (Sigma, MO, USA), 50 *μ*M l-ascorbic acid 2-phosphate (Sigma, MO, USA), 10 ng/ml epidermal growth factor (PeproTech, NJ, USA), and 1 ng/ml platelet-derived growth factor-BB (PeproTech, NJ, USA). Cells were incubated at 37°C and 5% CO_2_. When cells reached 70–80% confluence, they were detached with HyQTase (Thermo, Logan, UT) and replated at a ratio of 1 : 4.

### 2.2. Cytokines and Inhibitors

Cell cultures were starved for 16–18 hours in serum-free DMEM containing 0.1% bovine serum albumin and then treated with 2 *μ*g/ml IL-1*β* inhibitor IL-RA (PeproTech, NJ, USA) for 2 hours prior to cytokine stimulation. The MMP inhibitors TIMP1 and TIMP2 (PeproTech, NJ, USA) and MMP-1 inhibitor GM6001 (Merck, Darmstadt, Germany) were added to cell cultures 2 hours prior to IL-1*β* stimulation at concentrations of 45 nM, 45 nM, and 50 nM, respectively. 100 nM PAR1 inhibitor SCH79797 (Axon Medchem, Groningen, Netherlands) was added to cell cultures 2 hours before stimulation as previously described. At the indicated time, cells were incubated for 12–48 hours with 100 ng/ml human recombinant IL-1*β* (PeproTech, NJ, USA) in the continued presence of these inhibitors.

### 2.3. Cell Viability Assay

Cells were plated in 24-well plates in serum-free DMEM containing 0.1% BSA for 16 hours and stimulated with 0–500 ng/ml human recombinant IL-1*β* for 18 hours. PrestoBlue™ cell viability reagent was added directly to cells in the culture medium and incubated for 30 minutes at 37°C. The results were detected using multimode microplate readers (Infinite 200, Tecan).

### 2.4. MTT Assay

Cells were plated in 96-well plates in serum-free DMEM containing 0.1% BSA for 12–16 hours and stimulated with 100 ng/ml human recombinant IL-1*β* for 36 hours. MTT assay reagents (3-(4,5-dimethyl-2-thiazolyl)-2,5-diphenyl-2*H*-tetrazolium bromide; Serva, Heidelberg, Germany) were added directly to cells in culture medium and incubated 4 hours at 37°C, and then DMSO (Sigma, MO, USA) was added for 2 hours. The results were detected using multimode microplate readers (Infinite 200, Tecan).

### 2.5. Microarray Analysis

Total RNA was isolated from mesenchymal stem cells with or without IL-1*β* stimulation and IL-1RA stimulation. The RNA quality was checked by RNA electrophoresis before hybridizing with human genome U133 2.0 array chip (Affymetrix). The cDNA detection and raw data were obtained from the National Yang-Ming University VYM Genome Research Center.

### 2.6. Quantitative Real-Time Polymerase Chain Reaction

After stimulation, cells were harvested and the total RNA was extracted using the TriPure isolation reagent (Bioline, London, UK) according to the manufacturer's instructions. RNA was converted to cDNA with the Tetro cDNA synthesis kit (Bioline, London, UK). The following oligonucleotides were used for each gene: MMP-1 forward primer 5′-GATGGACCTGGAGGAAATCTTG-3′ and MMP-1 reverse primer 5′-TGAGCATCCCCTCCAATACC-3′ and GAPDH forward primer 5′-GGAGTCAACGGATTTGGTCGTA-3′ and GAPDH reverse primer 5′-GGCAATATCCACTTTACCAGAGT-3′.

Gene expression was analyzed by quantitative real-time PCR using SensiFAST CYBR Hi-ROX system (Bioline, London, UK), and each reaction was repeated in triplet.

### 2.7. MMP-1 Enzyme-Linked Immunosorbent Assay

After 48 hours of stimulation with IL-1*β*, the condition medium was collected. Quantitation of MMP-1 protein expression and activity was performed using RayBio human MMP-1 ELISA kit (RayBiotech, GA, USA). The results were detected at a wavelength of 450 nm using a spectrophotometer reader (ND-1000, NanoDrop).

### 2.8. Western Blotting

Cells were washed with PBS and lysed by M-PER mammalian protein extraction reagent (Thermo, IL, USA) with Halt protease inhibitor cocktail (Thermo, IL, USA) and were then centrifuged at 14,000*g* for 10 min at 4°C to collect the precleared cell extracts. Protein concentration was determined with the Coomassie Plus (Bradford) protein assay reagent (Thermo, IL, USA) using multimode microplate readers (Infinite 200, Tecan). Protein samples were resolved by 10% sodium dodecyl sulfate-polyacrylamide gel electrophoresis (SDS-PAGE) and transferred to polyvinylidene fluoride membranes (Merck, Darmstadt, Germany). Membranes were blocked in 10% fish gelatin blocking buffer (Amresco, OH, USA) for 1 hour and then incubated with the anti-human active form of PAR1 primary antibody (Sigma, MO, USA) at 1 : 2500 dilution and ERK 1/2 and phospho-ERK 1/2 primary antibodies (Cell Signaling Technology, MA, USA) at 1 : 2500 dilution at 4°C overnight. The blots were washed with Tris-buffered saline with Tween 20 (TBST) and incubated with goat anti-rabbit secondary antibody for 1 hour at room temperature. Membranes were washed and then detected by an enhanced chemiluminescence substrate using a luminescence imaging system (LAS 4000, GE, USA).

### 2.9. MMP-1 siRNA Oligonucleotides and Negative Control siRNA Transfection

MMP-1 Silencer Select predesigned siRNA (s8847 and s8848, Ambion, Austin, USA) and Silencer Select negative control no. 1 (Ambion, Austin, USA) were used to downregulate MMP-1 expression in cells. Cells were plated in 6-well plates for 24 hrs prior to transfection with 5 nM of MMP-1-specific siRNA or negative control siRNA using lipofectamine RNAiMAX transfection reagent (Invitrogen, CA, USA).

### 2.10. Wound Healing Assay

Cells were seeded at cell culture inserts (ibidi, Planegg, Germany) in 24-well plates and cultured until confluent. Cells were then starved in serum-free DMEM containing 0.1% BSA for 16–18 hours before adding inhibitors. After incubation with inhibitors IL-1RA (2 *μ*g/ml), TIMP1/2 (45 nM), GM6001 (50 nM), and SCH79797 (100 nM) for 2 hours, the cell culture inserts from 24-well plates were taken out and treated with 100 ng/ml IL-1*β*. MMP-1 siRNA-transfected cell groups were seeded in 24-well plates and cultured until confluent before adding IL-1*β* (100 ng/ml). Cell migration was observed with an inverted microscope, and pictures were taken in the same field every 6 hours after stimulation for 24 hours.

### 2.11. *In Vitro* Invasion Assay

The invasion assay was performed in an 8.0 *μ*m pore size Matrigel invasion chamber (Corning, MA, USA). Matrigel (Corning, MA, USA) was thawed and liquefied on ice, and then 30 *μ*l of Matrigel was added to 24-well transwell inserts and solidified in a 37°C incubator for 30 minutes to form a thin gel layer. Cells were seeded in 6-well plates and cultured until confluent. Cell cultures were starved for 12–16 hours in serum-free DMEM containing 0.1% bovine serum albumin and then treated with IL-1RA (2 *μ*g/ml) for 2 hours prior to IL-1*β* stimulation. At the indicated time, cells were incubated for 36 hours with 100 ng/ml IL-1*β* in the continued presence of these inhibitors. Cells were then detached with HyQTase, and 15,000 cells in serum-free DMEM were loaded into the upper Matrigel chamber, and complete MSC medium was added to the lower chamber. After 18 hours of incubation at 37°C, nonmigrated cells in the upper chamber were removed with a cotton swab, and cells that had migrated to the lower chamber were fixed and stained with crystal violet (Sigma, MO, USA).

### 2.12. Statistical Analysis

Statistical analyses were performed using the SPSS software (version 16.0). Quantitation of real-time PCR, ELISA, and cell wound healing assay data was analyzed by Student's *t*-test. *P* values < 0.05 were considered statistically significant.

## 3. Results

### 3.1. IL-1*β* Stimulates Mesenchymal Stem Cell Migration and Invasion

**Figure 1:**
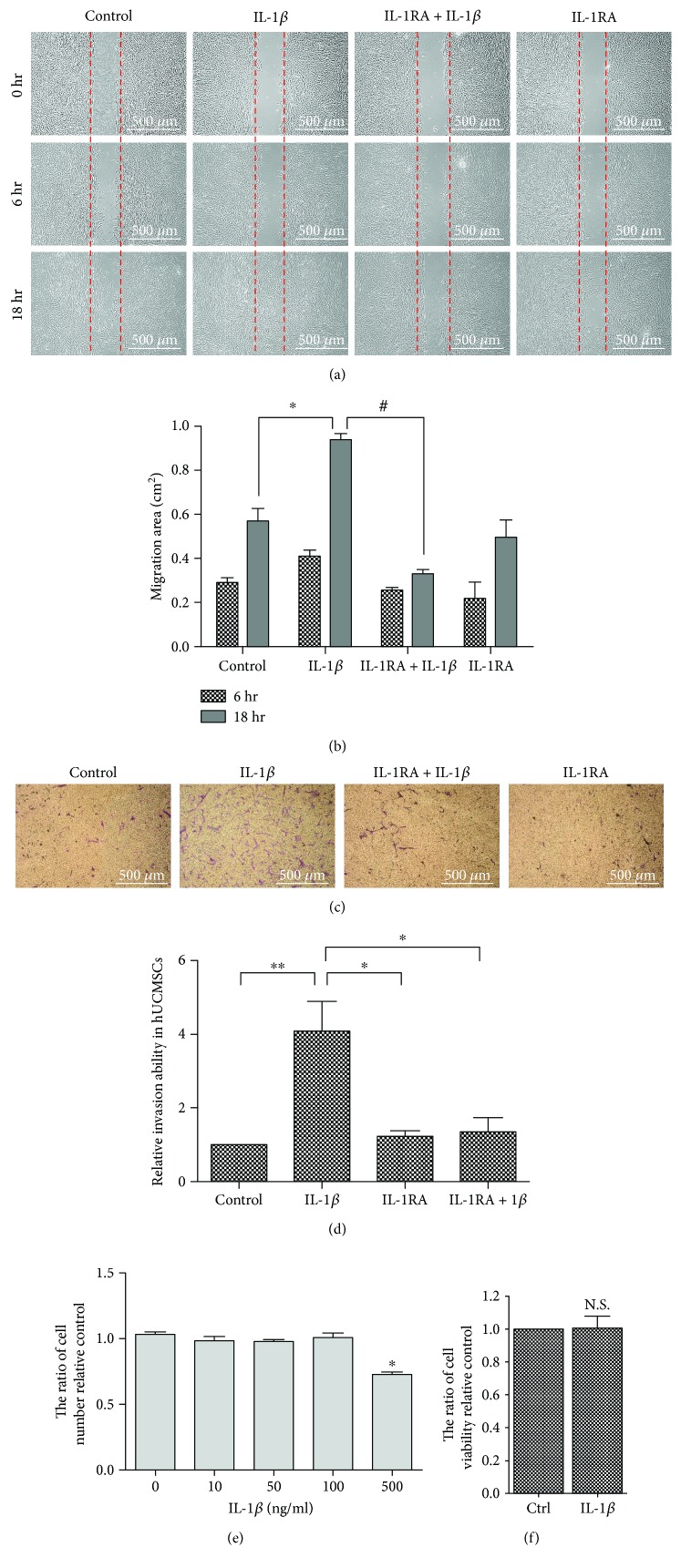
IL-1*β*-induced MSC migration and invasion. (a) Cell wound healing assay for IL-1*β*-stimulated mesenchymal stem cells in the presence or absence of 2 *μ*g/ml IL-1RA (IL-1*β* inhibitor) at 6 and 18 hours. Scale bars = 500 *μ*m. (b) Graph indicates the migration ability of stem cells into the wound area. Data are shown as mean ± SD (*n* = 3, ^∗^*P* < 0.05 versus control, ^#^*P* < 0.05 versus IL-1*β*). (c) Cell invasion assay; cultures were treated with IL-1*β* in the presence or absence of IL-1RA for 18 hours. (d) Quantitative data in invasion assay are presented as mean ± SD of triplicate samples. Data are shown as mean ± SD (*n* = 3, ^∗^*P* < 0.05, ^∗∗^*P* < 0.01). (e) Cell viability assay for IL-1*β* stimulation at different concentrations. Data were quantified by multimode microplate readers. Data are shown as mean ± SD (*n* = 3, ^∗^*P* < 0.05). (f) MTT cell proliferation data for IL-1*β* stimulation. Data were quantified by multimode microplate readers. Data are shown as mean ± SD (*n* = 3).

In vitro migration was performed using the wound healing assay. The results showed that 100 ng/ml IL-1*β* enhanced stem cell migration, whereas IL-1*β* antagonist IL-1RA could inhibit IL-1*β*-induced cell migration after treatment with IL-1*β* for 18 hours (Figures 1(a) and 1(b)). Matrigel-coated transwell invasion assay showed that while IL-1*β* enhanced mesenchymal stem cell invasion, IL-1RA could inhibit IL-1*β*-induced MSC invasion ability (Figures 1(c) and 1(d)). The cell viability assay indicated that IL-1*β* at a concentration of 500 ng/ml showed signs of cytotoxicity. Cell viability showed no significant change in the 100 ng/ml IL-1*β* group treated for 18 hours compared to the control group (Figure 1(e)). The MTT cell proliferation assay showed no significant change in IL-1*β*-treated MSCs in comparison to the control group (Figure 1(f)). These results indicated that IL-1*β*-induced stem cell migration and invasion was not affected by cell proliferation.

### 3.2. IL-1*β* Promotes MMP-1 Expression in Mesenchymal Stem Cells

**Figure 2:**
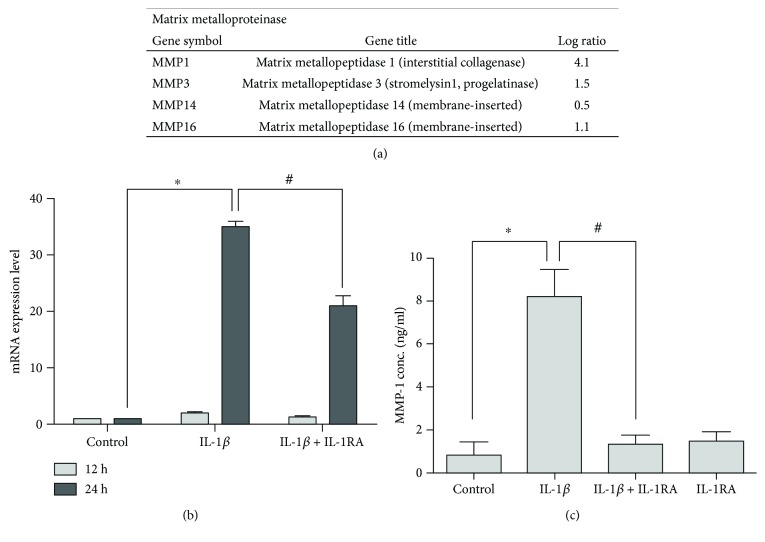
IL-1*β*-induced MMP-1 expression. (a) DNA microarray-based screening for the expression of MMPs with IL-1*β* stimulation for 24 hours in mesenchymal stem cells. (b) Quantitation of changes in the gene expression of MMP-1 detected by real-time PCR after IL-1*β* inhibitor IL-1RA (2 *μ*g/ml) pretreatment and stimulation with IL-1*β* for 24 hours. Data are shown as mean ± SD (*n* = 3, ^∗^*P* < 0.05 versus control, ^#^*P* < 0.05 versus IL-1*β*). (c) MMP-1 protein expression was measured using ELISA, pretreated with IL-1RA at concentration of 2 *μ*g/ml, and stimulated with IL-1*β* for 48 hours. Data are shown as mean ± SD (*n* = 3, ^∗^*P* < 0.05 versus control, ^#^*P* < 0.05 versus IL-1*β*).

To identify the molecular pathways involved in the migration of stem cells, gene expression profiles of IL-1*β*-treated cells and nontreated cells were determined by cDNA microarray using human genome U133 2.0 array (Affymetrix). Array results were analyzed, and we found that MMP-1 was upregulated significantly in mesenchymal stem cells treated with IL-1*β* (Figure 2(a)). The mRNA expression of MMP-1 was confirmed by quantitative real-time PCR.  Figure 2(b) shows that the levels of MMP-1 transcript in IL-1*β*-treated cells were higher than those in nontreated cells after 24 hours. To confirm that the MMP-1 expression was mediated by IL-1*β*, we pretreated the cells with IL-1*β* inhibitor IL-1RA. Results indicated that this inhibitor significantly suppressed IL-1*β*-induced MMP-1 expression. These findings are consistent with our microarray data.

To further confirm the expression of MMP-1 in stem cells treated with IL-1*β*, levels of MMP-1 protein expression were quantified using an ELISA. As shown in Figure 2(c), MMP-1 expression was significantly higher in IL-1*β*-treated stem cells compared with that in the control group after 48 hours. IL-1*β*-induced MMP-1 expression was inhibited by IL-1RA (Figure 2(c)). Taken together, these results indicate that IL-1*β* promotes MMP-1 expression in mesenchymal stem cells.

### 3.3. IL-1*β*-Mediated Cell Migration Depends on MMP-1 Secretion

**Figure 3:**
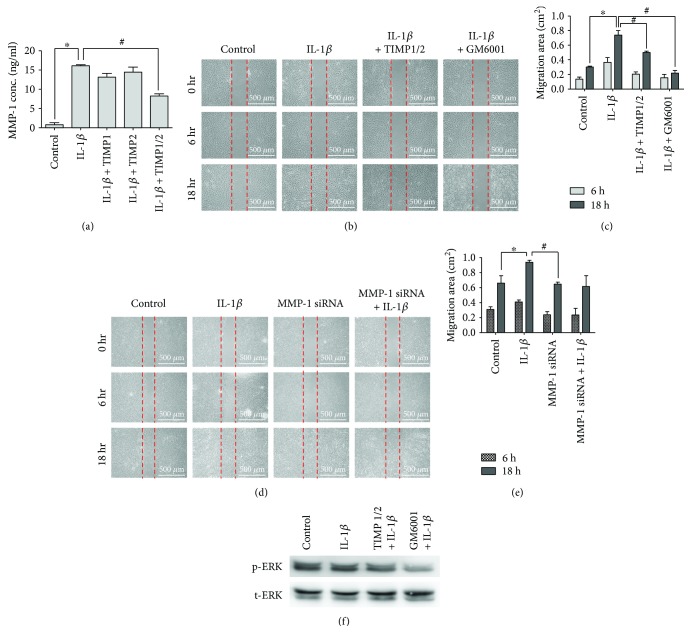
MMP-1 is required for IL-1*β*-induced MSC migration. (a) MMP-1 protein expression measured by ELISA. Pretreatment with both TIMP1 and TIMP2 together at a concentration of 45 nM reduced MMP-1 concentration in the culture. Data are shown as mean ± SD (*n* = 3, ^∗^*P* < 0.05 versus control, ^#^*P* < 0.05 versus IL-1*β*). (b) Cell wound healing assay. Cultures were treated with MMP inhibitors TIMP1 and TIMP2 and MMP-1 inhibitor GM6001 at concentrations of 45 nM, 45 nM, and 50 nM, respectively, as indicated. Scale bars = 500 *μ*m. (c) Quantitative graph showing the migration ability of stem cells into the wound area at 6 and 18 hours. Data are shown as mean ± SD (*n* = 3, ^∗^*P* < 0.05 versus control, ^#^*P* < 0.05 versus IL-1*β* at 18 hours). (d) Cell wound healing assay for IL-1*β*-stimulated MSCs after MMP-1 siRNA transfection (scale bars = 500 *μ*m). (e) Quantitative graph showing the migration ability of MMP-1 siRNA-transfected stem cells into the wound area at 6 and 18 hours. Data are shown as mean ± SD (*n* = 3, ^∗^*P* < 0.05 versus control, ^#^*P* < 0.05 versus IL-1*β* at 18 hours). (f) Western blot results of the phosphorylated ERK 1/2 from the lysates of cells pretreated with TIMP1/2 and GM6001.

To examine whether the observed mesenchymal stem cell migration ability was influenced by the expression of MMP-1, MMP inhibitors TIMP1 and TIMP2, MMP-1 inhibitor GM6001, and MMP-1 siRNA transfection were used in this experiment. The results showed that stem cell cultures treated with both TIMP1 and TIMP2 simultaneously inhibited MMP-1 protein expression (Figure 3(a)). Wound healing assay indicated that cells treated with TIMP1 and TIMP2 together attenuated IL-1*β*-induced cell migration (Figures 3(b) and 3(c)). Pretreatment with another MMP-1 inhibitor GM6001 also decreased IL-1*β*-induced cell migration (Figures 3(b) and 3(c)). MMP-1 siRNA transfection of MSCs decreased IL-1*β*-induced cell migration (Figures 3(d) and 3(e)). Western blot showed that MSCs pretreated with TIMP1/2 and GM6001 attenuated IL-1*β*-induced ERK 1/2 phosphorylation (Figure 3(f)), suggesting that IL-1*β*-induced MMP-1 expression involved the activation of ERK 1/2 signaling cascades.

### 3.4. MMP-1 Which Was Mediated by IL-1*β* Regulates the Activation of PAR1

**Figure 4:**
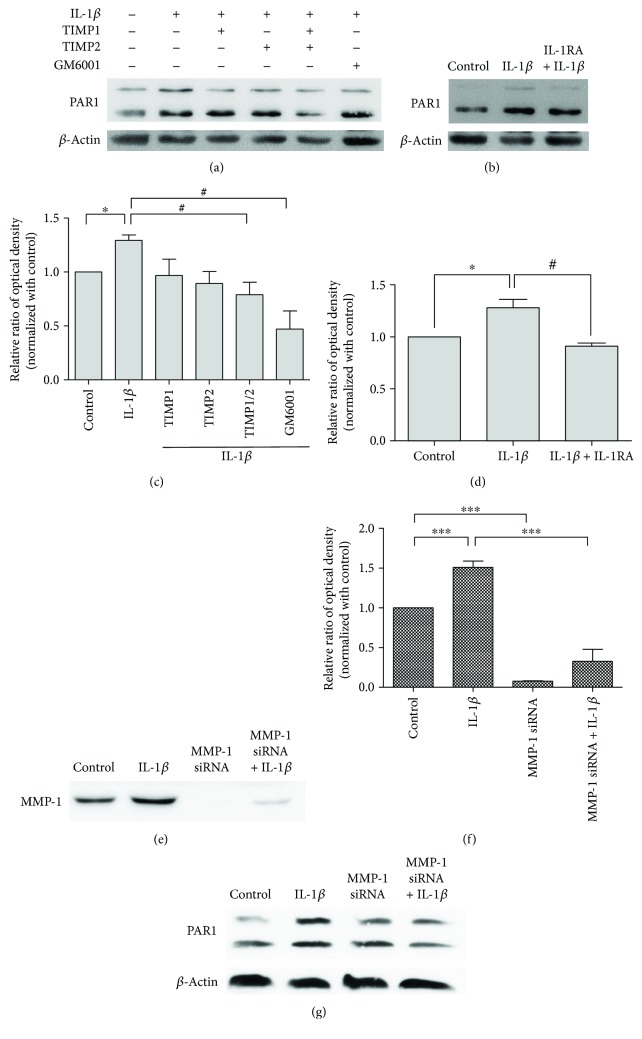
Effect of IL-1*β* receptor antagonist, MMP-1 inhibitor, and MMP-1 siRNA on IL-1*β*-induced PAR1 expression. (a) Example of Western blot results of the active form of PAR1 (46 kDa) from the lysates of cells pretreated with TIMP1, TIMP2, and GM6001. (b) Example of Western blot results of PAR1 expression from the culture treated with IL-1*β* and IL-1RA. (c and d) Quantitative graphs of the Western blot results of the active form of PAR1 protein expression of (a) and (b), respectively. Data are shown as mean ± SD (*n* = 3, ^∗^*P* < 0.05 versus control, ^#^*P* < 0.05 versus IL-1*β*). (e) Example of Western blot results of MMP-1 released in the medium after siRNA transfection with/without IL-1*β* stimulation. (f) Quantitative graphs of the Western blot results of MMP-1 released in the medium of (e). Data are shown as mean ± SD (*n* = 3, ^∗∗∗^*P* < 0.001). (g) MSCs transfected with MMP-1 siRNA attenuated PAR1 expression.

In recent studies, the G-protein-coupled receptor PAR1 has been found to be cleaved and activated by MMP-1, which promotes cancer cell migration and invasion [20, 34]. To investigate whether PAR1 plays a role in IL-1*β*-induced MMP-1 expression in mesenchymal stem cells, PAR1 Western blotting analysis was performed in cultures pretreated with MMP inhibitors TIMP1 and TIMP2, MMP-1 inhibitor GM6001 (Figure 4(a)), and IL-1RA (Figure 4(b)). The results showed obvious decrease in the expression of the active form of PAR1 (Figures 4(c) and 4(d)). MMP-1 siRNA transfection of MSCs knock downs by at least 80% IL-1*β*-induced MMP-1 release in the medium (Figures 4(e) and 4(f)). The results indicated that MMP-1 siRNA transfection revealed high transfection efficiency. In the MMP-1 siRNA transfection of MSCs, the Western blot showed that IL-1*β*-induced PAR1 expression was attenuated (Figure 4(g)). These findings indicate that MMP-1 induced by IL-1*β* regulates the activation of PAR1.

### 3.5. IL-1*β*-PAR1 Signaling Axis Influences Stem Cell Migration

**Figure 5:**
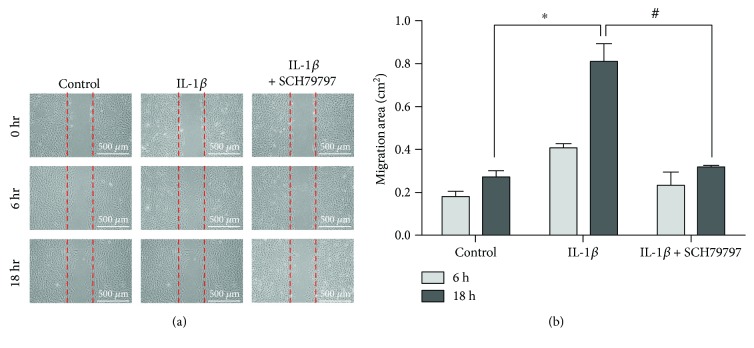
Effect of PAR1 inhibitor on IL-1*β*-induced MSC migration. (a) Cell wound healing assay for PAR1 inhibitors; SCH79797 pretreatment at a concentration of 100 nM. Scale bars = 500 *μ*m. (b) Quantitative analysis of the migration ability of stem cells treated with IL-1*β* and IL-1*β* + SCH79797. Data are shown as mean ± SD (*n* = 3, ^∗^*P* < 0.05 versus control, ^#^*P* < 0.05 versus IL-1*β*).

To determine IL-1*β*-stimulated MSC migration, wound healing assays were performed in cultures pretreated with PAR1 inhibitor SCH79797. Figures 5(a) and 5(b) show that IL-1*β*-induced stem cells were attenuated when SCH79797 was added to the cells, thus demonstrating that IL-1*β*-induced mesenchymal stem cell migration is mediated by interaction with PAR1.

## 4. Discussion

Mesenchymal stem cells have been reported for homing and long-term engraftment into the appropriate target tissue [35]. During the past decade, it has been confirmed that inflammation cytokines or growth factors are migratory cues in mesenchymal stem cell migration to the injured region. These cues include stromal cell-derived factor- (SDF-) 1*α*, transforming growth factor- (TGF-) *β*1, monocyte chemotactic protein- (MCP-) 1, and tumor necrosis factor- (TNF-) *α* [2, 6].

In this study, we have demonstrated that proinflammation cytokine IL-1*β* plays a role in mesenchymal stem cell migration. IL-1*β* enhances stem cell migration but does not influence cell proliferation. Some studies suggested that IL-1*β* may activate downstream protein kinase cascades, leading to the expression of inflammatory proteins [8]. Furthermore, it has been demonstrated that IL-1*β* can stimulate lymphocyte and eosinophil cell migration [9, 10]. Using DNA microarray analysis, our results showed that MMP-1 is significantly upregulated in IL-1*β*-treated stem cells. This was confirmed by the ~35-fold higher level of MMP-1 transcripts in the IL-1*β*-treated stem cells compared with that in nontreated cells using real-time PCR. The mRNA expression of MMP-1 was further supported by ELISA: stem cells treated with IL-1*β* were shown to secrete substantial amounts of MMP-1 into the culture supernatants. MMP-1 is a kind of matrix metalloproteinase which degrades collagen type I, and it has been reported that the condition medium of TGF-*β*1-treated adipose-derived stem cells can increase fibroblast MMP-1 expression and promote cell migration [36].

Recently, Boire et al. found that PAR1 is a MMP-1 receptor which promotes the invasion and tumorigenesis of breast cancer cells in vitro and in vivo [20]. Shi et al. demonstrated that blocking PAR1 cleavage and activation inhibits the invasion and chemotaxis of prostate cancer cells [37]. In our study, the active form of PAR1 protein expression was decreased by IL-1*β* inhibitor IL-1RA. A recent study reported the presence of GM6001 in stem cell culture block cell migration in collagen-based invasion assay [38]. It seems that MMP-1 plays a role in extracellular matrix degradation and further affects cell movement directly. Using MMP inhibitors TIMP1 and TIMP2, MMP-1 inhibitor GM6001, and MMP-1 siRNA transfection, we showed that IL-1*β*-induced PAR1 expression was inhibited. Moreover, using the PAR1 inhibitor SCH79797, we showed that blocking MMP-1-PAR1 interaction significantly reduced the migration ability mediated by IL-1*β* in stem cells. Thus, it appears that the level of MMP-1 expression and the specific interaction of MMP-1 with PAR1 determine the migration ability of stem cells. Additionally, Ho et al. [21] demonstrated that MMP-1 plays an important role in the migration function of mesenchymal stem cells, operating through the MMP-1-PAR1 signaling axis.

**Figure 6:**
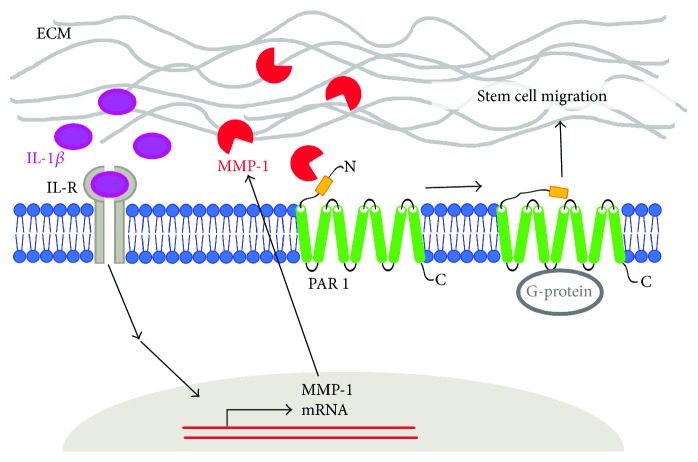
A schematic diagram depicting the proposed role of IL-1*β* signaling pathway in MSC migration. The process of cell migration is initiated by IL-1*β*-induced expression of MMP-1 in MSCs. The resultant increase of PAR1 activity causes cell migration.

In the future, the homing function of stems cells may provide the basis for the important clinical application of stem cells as a cellular vehicle for anticancer therapeutics in tumors [39] and regenerative medicine. Several factors that affect the homing potential of stem cells should be considered, including the ability of mesenchymal stem cells to respond to migratory stimuli, physiological barriers blocking stem cell migration, and inflammatory microenvironments of the body. Given the characteristics of stem cells, a range of therapeutic strategies can be explored to augment homing capability [2]. In conclusion, the results of this study suggest that the IL-1*β*-mediated expression of MMP-1 is associated with stem cell migration and that the MMP-1-PAR1 signaling axis is involved in IL-1*β*-mediated MMP-1 expression in promoting mesenchymal stem cell migration (Figure 6).
